# A retrospective records review comparing the care of patients who either avoided or were admitted to an ICU following a ward-based deterioration event

**DOI:** 10.1016/j.iccn.2025.104064

**Published:** 2025-10

**Authors:** J. Ede, H. Pickworth, B. Kent, P. Watkinson, R. Endacott

**Affiliations:** a Oxford University Hospital NHS Foundation Trust, Oxford, United Kingdom; b School of Nursing and Midwifery, University of Plymouth, Plymouth, United Kingdom; c University of Oxford, Nuffield Department of Clinical Neurosciences, Oxford, United Kingdom; d National Institute for Health and Care Research, 60 Cannon St, London, United Kingdom

**Keywords:** Clinical deterioration, Wards, Early Warning Scores

## Abstract

**Objectives:**

To identify escalation success factors documented in care records of patients who triggered an Early Warning Score ≥ 7 in the ward, avoided an Intensive Care Unit admission and survived and compare these with ward patients who triggered an Early Warning Score ≥ 7, went to intensive care and died during their admission.

**Methods:**

A multi-site, retrospective records review was conducted on 340 survivors and 50 non-survivors who were either admitted to, or who avoided intensive care.

**Results:**

Non-survivors of deterioration tended to be older, earlier into their hospital admission, and had a greater number of co-morbidities at the time of their trigger event. Overall, superior care was observed in non-survivors when triangulating quality of care scores and escalation care quality metrics (escalation compliance, hourly observations, and medical re-evaluation). Survivors avoided an Intensive Care Unit admission through responding to ward management or being referred to a specialist team. However, 9.7 % (33/340) of survivors were still triggering at the time of discharge, and 54 % of these had either Covid-19 or a long-term cardiorespiratory condition.

**Conclusions:**

This study found differences in how clinical staff responded to patient deterioration between survivors and non-survivors. Although non-survivors received higher-rated care and met more escalation quality indicators, their poorer outcomes were likely influenced by more severe underlying conditions. Despite both patient groups having comparable scores, staff appeared to make nuanced judgments factoring in clinical concerns not captured by the score alone (success factor).

**Implications for Clinical Practice:**

Despite generating the same warning score values, there is wide variation in true patient acuity that only clinical staff can discriminate, and escalation protocols alone may not be advanced enough to address this subtlety.

## Introduction

Failures to detect and manage patient deterioration are common in healthcare settings worldwide [1,2]. Adverse events, such as cardiac arrests, are often preceded by vital sign abnormalities for up to 48 h prior to the event, highlighting the potential for early remedial interventions [3]. These events highlighted a need for earlier recognition of patient deterioration leading to the development of Early Warning Scores (EWS), that use patient’s physiological data to generate an aggregate score [4,5] to facilitate clinical decision making [6]. Scores are calculated based on the degree of abnormality in a patient’s physiological parameters and include respiratory rate, oxygen saturation, systolic blood pressure, heart rate, level of consciousness and in some tools, oxygen requirements [6,7]. Higher score thresholds represent a patient’s risk of deterioration or death and cumulative scores of 5 or above likely indicate urgent physiological derangement and high-risk [6,[8], [9], [10]].

Despite many studies examining sentinel Failure to Rescue (FTR) events and the implementation of EWS systems, there is little evidence of strong safety improvements [11]. Evidence in this field often comes from safety investigations, such as Root Cause Analysis, that aim to identify and address contributory factors through system changes to reduce the likelihood of reoccurrence [12]. However, this traditional approach makes assumptions about cause-and-effect relationships within a complex healthcare system, and assume safety originates from the reliability of components [13]. Traditional reductionist approaches may therefore have hindered our understanding of escalation problems and potential solutions [14]. For instance, recognising patient deterioration and detecting problems exist, are complex processes that necessitate clinical expertise [15].

Many other safety critical industries (rail, nuclear) examine both adverse events (which are rare) and events with good outcomes [16]; termed Safety-I and Safety-II approaches respectively [17]. If healthcare modelled effective processes by understanding why some patients have good outcomes, then further care improvements could be implemented. Identifying the factors that are part of a successful care escalation (success factors) in patients who became acutely unwell in the ward but avoided further deterioration or an Intensive Care Unit (ICU) admission could underpin this.

The aims of this study were to i) identify escalation success factors documented in care records of patients who triggered an Early Warning Score (EWS) ≥ 7 in the ward, avoided ICU and survived and ii) compare these with ward patients who triggered a EWS ≥ 7, went to ICU and died.

## Methods

### Research design

This is a multi-site, retrospective records review study comparing the quality of care between survivors and non-survivors of critical ward deterioration events to identify escalation success factors. This study was granted ethical approval from Queens Square London Research Ethics Committee (HRA-20HRA/3828), the Confidentiality Advisory Group (CAG) to review patient’s records (CAG-20CAG0106) and registered with ISRCTN 38850. The paper has been prepared and reported according to the STROBE guidelines (Supplementary File 1) and the published study protocol [18].

#### Care record reviews process

The Structured Judgement Review (SJR) process is a standardised approach to examining patient care used within the National Health Service (NHS) [19]. This method has also been applied to international and UK-based studies examining the care of certain patient groups [20,21]. Two phases of care record reviews were conducted in this study. All care records for 390 consecutive survivors and non-survivors, who deteriorated within the ward environment, were examined to grade quality of care and escalation, and to extract deterioration event data pertinent to the aims of the study. A sub-set of 40 in-depth reviews were then conducted (>10 % of the total number of reviews) to fully examine patient care before, during and after the deterioration event. In-depth reviews were discussed between both researchers and the research team. Reviews were completed by two researchers (HP and JE) who were both trained in the care record review method [22].

#### Quality of care judgements

The SJR review process advises that quality of care is defined through *‘judgements about the quality of care the patient received and whether it was in accordance with current good practice (for example, your professional standards or your professional perspective*).*’* [[15], p5]. The quality of care for this study was judged based on multiple care domains, such as documentation, timeliness of vital signs and medical re-reviews following a trigger event. Care that was judged to be ‘Poor’ was given a score of 1 and care that was deemed ‘Excellent’ given a score of 5. This approach was consistent with the published SJR review methods [19,23] and available NHS training.

#### Escalation metrics

Predefined quality of escalation care metrics, defined in Supplementary File 2, formed part of the reviewers’ care score judgements. These represent key elements of the escalation of care process and were identified through current literature, national guidelines and previous observations of escalation events [24]. ‘Vital signs observations being completed within 1 h’ and ‘referral to critical care’ were drawn from national escalation guidelines [25]. Other judgements, such as ‘Sepsis 6′ compliance [26] and relative involved with care, were developed within the research team.

#### Factors affecting avoidance of ICU

The predominant factors that contributed to survivors avoiding an ICU admission were captured. All non-survivors were ultimately admitted to ICU, so this data capture was not applicable. Categories for ICU admission avoidance were based on the work of Malycha and colleagues [27] who documented patients as responding to ward-based treatments, referred to specialist team, supported by ICU/Outreach, or treatment limitations. The categories were adapted for this work to further include ‘trigger unresolved at discharge’.

### Sample selection

Consecutive survivors and non-survivors were identified through the hospital information system and electronic records database between October 2019 and September 2020. We defined clinically unwell as a EWS score of ≥7 as this would warrant an ICU review or possible admission and represents a point of significant patient risk.

#### Inclusion

Ward patients were included if their EWS score was ≥7 or higher at any point during their admission. This trigger event must have occurred while they were ward in-patients, and they must not have been admitted to the ICU and survived to discharge. We also reviewed medical records where patients became unwell within the ward, were admitted to ICU and died at any point during their admission (in ICU or once discharged) to give a more comprehensive understanding of care.

#### Exclusion

Patient notes were excluded if they had an active Do Not Attempt Cardiopulmonary Resuscitation (DNACPR) order, were not within the ward environment at the time of trigger or had incomplete notes.

### The setting

This study was conducted across two separate NHS organisations in England. Site A, a group of three tertiary referral hospitals and one smaller hospital with almost 1500 beds, serving a population of over 600,000. The total hospital ICU bed capacity was approximately 48 beds with no established Critical Care Outreach Team. Site B, the main provider of acute hospital services for the population of approximately 500,000 people, with over 800 beds and a well-established, nurse-led critical care outreach team together with an ICU capacity of 16 beds.

### Data collection tool

Descriptive data were collected using a piloted case report from built within a spreadsheet (Supplementary File 3). The original SJR form consisted of time periods (including an end of life period), care scores, assessments of problems in care which were pre-specified (e.g. Problem in assessment, investigation or diagnosis) and sections for free text. The original SJR tool also gives an avoidability of death judgement score. This study was not designed to assess avoidability of death and therefore no scores were given. Our tool was an adaptation of the original, in order to collect a broader range of patient, illness and demographic data. Patient care data were reviewed and collected centring around defined time points including 24 h pre-trigger, 24 h post-trigger and >24 h post-trigger (subsequent care period until three subsequent EWS triggers of ≤3).

### Data analysis

The quantitative data from the care record were analysed using SPSS ® (IBM Corp. Released 2020. IBM SPSS Statistics for Windows, Version 27.0. Armonk, NY: IBM Corp). Analyses compared survivors and non-survivors (Supplementary File 4). Descriptive statistics were conducted reporting median, interquartile ranges and percentages where appropriate. Care score confidence intervals for percentages were calculated with the Clopper-Pearson method. To assess interrater reliability a Weighted Kappa was calculated, and significant agreement was assumed with a result of >0.64 based on a previous notes review study [28].

## Results

A total of 390 care records were reviewed including 340 survivors and 50 non-survivors.

### Patient demographics

Survivors and non-survivors matched in terms of gender and frailty scores, but non-survivors tended to be older and had a greater number of co-morbidities at the time of their trigger event. Full patient characteristics are detailed in Table 1.

**Table 1:** Patient characteristics.

Characteristic	EWS ≥ 7 Survivors n = 340		EWS ≥ 7 Non-survivors n = 50	
Age median (IQR)	58 (18)		64 (17)	
Female n (%)	142 (41.8)		21 (42)	
LOS median (IQR)	7.2 (7.3)		9 (8.9)	
Charlson Co-morbidity Index median (IQR)	2 (3)		4 (4)	
Clinical Frailty Scale median (IQR)	4 (3)		4 (2)	
Hospital Admission Type n (%)				
Emergency	299 (88)		48 (96)	
Elective	41 (12)		2 (4)	
Admitting Team n (%)				
Medical	216 (63.5)		36 (72)	
Surgical	105 (30.9)		13 (26)	
Trauma	19 (5.6)		1 (2)	
Commonest trigger causes				
Sepsis	22.6		30.0	
Community Acquired Pneumonia	9.1		8.0	
Hospital Acquired Pneumonia	7.9		8.0	
Covid-19 Infection	9.4		12.0	
Covid-19 Positive n (%)*	51 (15)		7 (14)	

*Patients may be infected with Covid-19, but this was not the cause of trigger episode.

### Trigger event characteristics

The median number of days from admission to trigger event was longer in survivors (2.1 days, IQR 3.2) versus non-survivors (1.2 days, IQR 3.6). Median trigger scores (the first documented episode of a warning score ≥ 7) for survivors tended to be lower (8, IQR 2) than that of non-survivors (9, IQR 3) (Supplementary File 4). A prevalence comparison of abnormal physiological parameters in the survivors and non-survivors’ group is detailed in Supplementary File 5.

### Quality of escalation care

Agreement between reviewers on care quality was strong (Weighted Kappa = 0.74, 95 % CI: 0.59–0.89) across all assessed scores. Median quality of care scores for survivors tended to be lower (3, IQR 1) than non-survivors (4, IQR 1). Overall, 85 % of Survivors and 96 % of non-survivors were judged to have had adequate care or above (Table 2).

**Table 2:** Overall quality of care scores.

Care score n (%)	EWS ≥ 7 Survivors n = 340 (%)	95 % CI	EWS ≥ 7 Non-survivors n = 50 (%)	95 % CI
1. Very poor care	0	0–1	1 (0.6)	1–11
2. Poor Care	51 (15)	11–19	3 (3.4)	1–16
3. Adequate care	125 (36.8)	32–42	15 (25.6)	18–45
4. Good care	136 (40.0)	35–45	31 (70.5)	47–75
5. Excellent Care	28 (8.2)	5–12	0	0–7

−–No data.

Survivors tended to be less frequently escalated and were less likely to have vital signs completed within 1 h than non-survivors (43.2 % v 72 %). Overall, non-survivors were judged to have better quality of documentation, medical reassessments within 4 h of trigger and a relative or next of kin involved with care. Quality of escalation metrics are detailed in Table 3.

**Table 3:** Assessed care metrics.

Care metric n (%)	Survivors (n = 340)	Non-Survivors (n = 50)
Escalated according to local policy	147/340 (43.2 %)	36/50 (72 %)
Vital signs observations checked within 1 h of trigger event	180/340 (52.9 %)	27/46* (54 %)
Sepsis 6 care bundle completed (for patients with Sepsis)	45/77 (57.1 %)	8/15 (53.3 %)
Missing Sepsis 6 element		
Lactate	9/32** (30 %)	1/7 (14.3 %)
Urine output	14/32 (47 %)	3/7 (42.9 %)
Blood cultures	6/32 (20 %)	2/7 (28.6 %)
O2	1/32	1/7 (14.3 %)
Intravenous fluids	1/32 (3 %)	−-
Antibiotics	−-	−-
Re-assessed by medical team within 4 h of trigger	163/340 (47.9 %)	45/50 (90 %)
Good quality of documentation	233/340 (68.5 %)	45/50 (90 %)
Relative involved with care	67/340 (19.7 %)	26/50 (52 %)
Review referral made to ICU	125/340 (37.8 %)	50/50 (100 %)

*4 patients in ICU within 1 h of observations.

**No data for 1 care record.

−- Not present in data set.

### ICU admission avoidance

Most survivors tended to avoid an ICU admission because they responded to ward-based interventions (250/340, 73.5 %). In 9.7 % of survivors (33/340), there was no resolution of the trigger event even at the point of discharge. Of the patients with unresolved trigger events 54 % had either Covid-19 (7/13, 21.2 %), a long term respiratory (COPD; 7/33, 21.2 %) or cardiac condition (CCF; 3/33, 9.1 %). Other than Outreach or ICU, specialist team referral was most commonly to anaesthetists, endocrine, respiratory and surgery. A full description of factors which contributed to the avoidance of an ICU admission in survivors are detailed in Table 4.

**Table 4:** Factors contributing to avoidance of an ICU admission in survivors.

Reason for ICU avoidance	Frequency (%)
Referred to specialist team	23 (6.8)
Responded to ward-based treatments	250 (73.5)
Supported by ICU/Outreach	32 (9.4)
Treatment limitations	2 (0.6)
Trigger unresolved at discharge	33 (9.7)

*Some patients may have been referred to ICU or Outreach, but this was not deemed the primary factor contributing to ICU avoidance.

## Discussion

This study reviewed 390 care records, comparing survivors and non-survivors of critical deterioration events. Our findings indicate that most deterioration events occurred among medical patients, followed by surgical and trauma patients which was consistent across both patient cohorts. Notably, survivors avoided an ICU admission primarily through effective ward management and specialist team referrals, although 9.7 % of survivors had unresolved trigger events at discharge. Non-survivors had higher trigger scores, more comorbidities, were older and earlier into their admission at the time of their critical event. Despite receiving higher quality care, as evidenced by superior care scores and escalation quality metrics, non-survivors’ more severe conditions likely contributed to their poor outcomes.

Our data highlights differences in staff responses to both cohorts, even when trigger scores were similar, and this may represent an escalation success factor. Differences to escalation practices may be influenced by clinical signals and staff judgments of patient risk. For example, at the time of their trigger event, non-survivors had higher median trigger scores and a greater prevalence of hypotension and hypothermia. These are strong predictors of mortality [29] and indicate a more significant deterioration event in our patient cohort. This was in contrast to survivors’ trigger events that occurred later into their admission and were predominantly resolved through ward-based treatments. It is unsurprising then, given the severity of the trigger event, that we also found that non-survivors had vital signs observations completed more frequently within 1 h than survivors. This finding supports those of other studies that identified an association between more frequent vital signs measurements and higher mortality odds [[30], [31], [32]]. Non-survivors received high resource care but continued to have a poor outcome.

The differences in care between both patient cohorts would indicate that staff recognised that the non-survivor group were in some way sicker and at a higher risk of an adverse event, which may also explain why compliance with escalation protocols was higher in the non-survivor group (72 %) compared to the survivor group (43.2 %). Lower than expected levels of escalation is a common and a recurring finding across the literature; a study in an Australian Emergency Department showed that nearly half of patients (47.6 %) requiring escalation were not escalated according to the local standard [33]. Another multi-site study found that only 42 % of clinically unwell patients meeting Rapid Response Team escalation criteria were actually escalated [29]. This pattern is common in healthcare settings. While some non-escalations may be due to error, the reasons behind many remain unclear.

An additional important finding from our study was that nearly 10 % of patients who survived still had an unresolved trigger event at discharge. The majority of these patients had long-term chronic conditions, which challenges the ability of EWS to discriminate effectively [34,35]. Whilst NEWS performs better than historical tools, it does perform poorly in certain populations such as chronic lung patients [35]. This phenomenon can create a significant workload for staff, especially when escalation actions are driven by warning score thresholds [36]. Our data, along with existing evidence, suggests that escalation should be (and is often) tailored to individual patient needs. Notably, while all patients in our study were screened with an EWS of ≥7 or higher, acuity varied within each individual score. Currently, only clinical staff can accurately assess this range of acuity within identical numerical values [37] and further reinforces the requirement for a highly skilled and specialised workforce.

## Limitations

The data presented in this work is from 2022, and whilst results should be viewed with caution, the learning generated from this study is likely still informative and applicable more generally. Limitations of the record review methods should be considered and not all care that is delivered is documented [38] and mitigations to reduce hindsight bias in the notes review process have been attempted with a second reviewer and regular data meetings. There is a gap in this study, in that it has only captured the traditional triggering patients and not those patients who were unwell and not triggering. Finally, some patients who triggered a EWS ≥ 7 may have improved with minimal hospital input as they had a better physiological reserve, and so not all patients who survived did so because of a true rescue event.

## Conclusion

Our study has described the cohorts of patients who either survived or died following their ward deterioration event. Our record reviews found fundamental differences in the escalation process between survivors and non-survivors, despite having similar EWS scores, indicating that staff are making nuanced judgements about patient risk. Our study suggests that clinical judgment remains crucial in distinguishing between patients who may recover on the ward and those requiring higher levels of care.

## Clinical trial registration

Reported in this manuscript is a sub-study of the larger mixed methods SUFFICE study. This was registered within the ISRCTN database (ISRCTN23769228).

## Compliance with ethical standards

We confirm that the study was conducted in accordance with relevant ethical guidelines and regulations, such as the Declaration of Helsinki for research involving human subjects.

## CRediT authorship contribution statement

**J. Ede:** Writing – original draft, Visualization, Validation, Supervision, Software, Resources, Project administration, Methodology, Investigation, Funding acquisition, Formal analysis, Data curation, Conceptualization. **H. Pickworth:** Writing – review & editing, Project administration, Methodology, Formal analysis, Data curation, Conceptualization. **B. Kent:** Writing – review & editing, Writing – original draft, Supervision, Resources, Project administration, Methodology, Investigation, Funding acquisition, Formal analysis, Data curation, Conceptualization. **P. Watkinson:** Writing – review & editing, Writing – original draft, Supervision, Software, Resources, Methodology, Investigation, Funding acquisition, Conceptualization. **R. Endacott:** Writing – review & editing, Writing – original draft, Visualization, Validation, Supervision, Software, Resources, Project administration, Methodology, Investigation, Funding acquisition, Formal analysis, Data curation, Conceptualization.

## Declaration of competing interest

The authors declare that they have no known competing financial interests or personal relationships that could have appeared to influence the work reported in this paper.

## Data Availability

The data that support the findings of this study are available from the corresponding author upon reasonable request.
