# Supplementary material for: A retrospective records review comparing the care of patients who either avoided or were admitted to an ICU following a ward-based deterioration event
Source: Intensive Crit Care Nurs. 2025 Oct;90:None. doi: 10.1016/j.iccn.2025.104064 (PMC12396344; doi:10.1016/j.iccn.2025.104064)
Supplement: Supplementary Data 2 [file mmc2.docx]

Supplementary File 2 Supplementary File Study Definitions

| EWS Trigger Event | The first EWS score that the patient has that is ≥7. This first EWS Trigger event may be higher than 7. |
| --- | --- |
| Resolution of Trigger Event | Care period demonstrating 3 subsequent triggers of <3 |
| Care periods used for data extraction | • 24 hours pre-trigger  • 24 hours post-trigger  •>24 hours post-trigger (subsequent care period until 3 subsequent triggers of <3) |
| Escalation quality care metrics | - Vital signs compliance - Compliance with local escalation policy - Sepsis 6 completion (for patients who have sepsis) |
| Acutely Unwell | Any patient with a EWS score of ≥7 which would warrant an ICU review or an admission |
| Quality of care judgements | All elements of a patient care record will be examined to make care judgements. The quality of care for this study was judged on multiple care domains such as documentation, timeliness of vital signs and medical re-reviews following a trigger event. Care scores were:   - Very poor care (1) - Poor Care (2) - Adequate care (3) - Good care (4) - Excellent Care (5) |
