# Supplementary figures and images for: A retrospective records review comparing the care of patients who either avoided or were admitted to an ICU following a ward-based deterioration event
Source: Intensive Crit Care Nurs. 2025 Oct;90:None. doi: 10.1016/j.iccn.2025.104064 (PMC12396344; doi:10.1016/j.iccn.2025.104064)

Supplementary File 3 Care Record Reviews Case Report Form

##
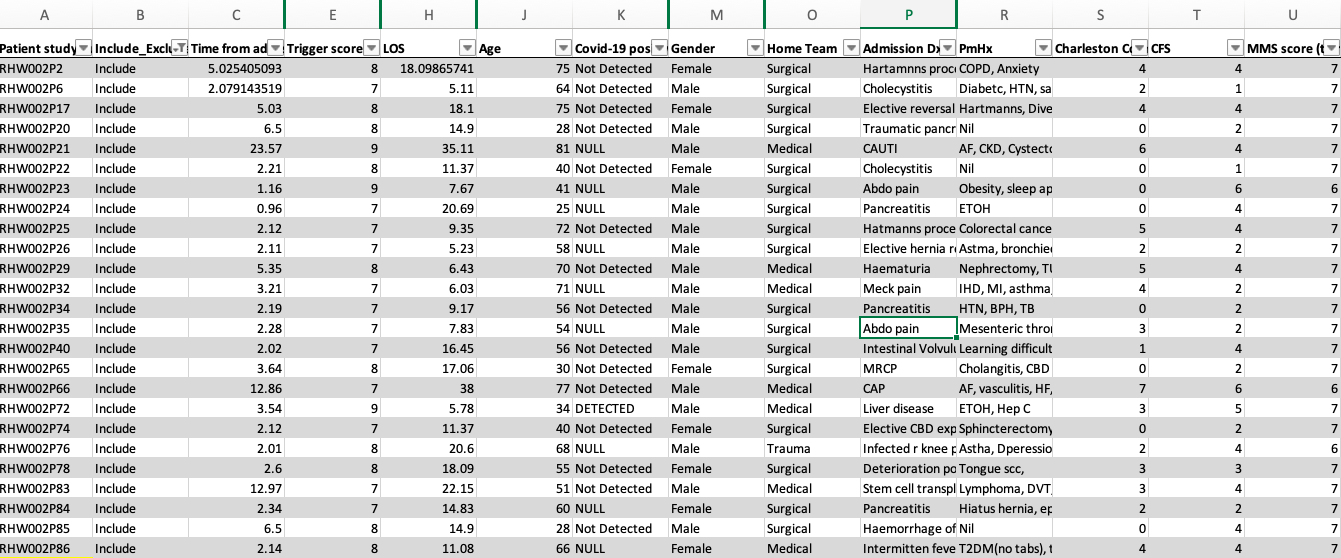

Supplement: Supplementary Data 3 [file mmc3.docx]
