# Supplementary material for: A retrospective records review comparing the care of patients who either avoided or were admitted to an ICU following a ward-based deterioration event
Source: Intensive Crit Care Nurs. 2025 Oct;90:None. doi: 10.1016/j.iccn.2025.104064 (PMC12396344; doi:10.1016/j.iccn.2025.104064)
Supplement: Supplementary Data 4 [file mmc4.docx]

Supplementary File 4 Statistical Analysis

### Patient Descriptive statistics


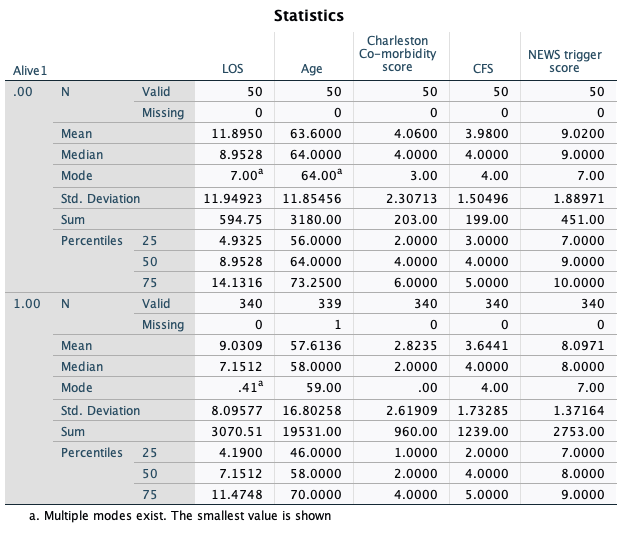


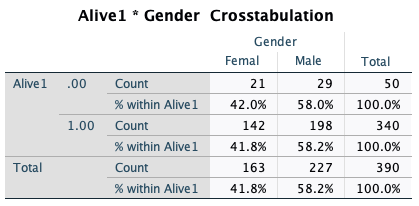


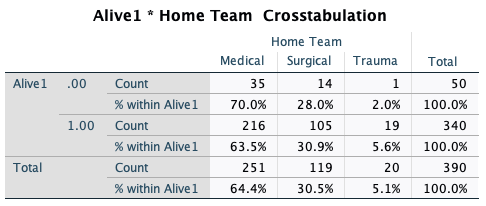


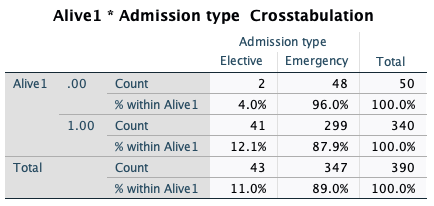


## Overall Quality of Care and Clopper-Pearson Confidence Intervals


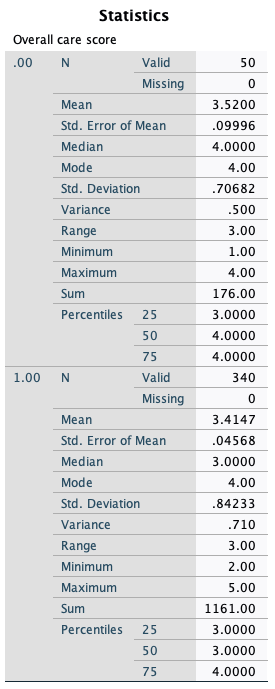


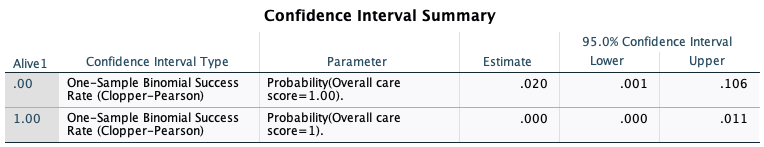


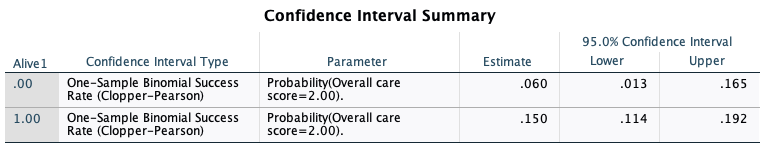


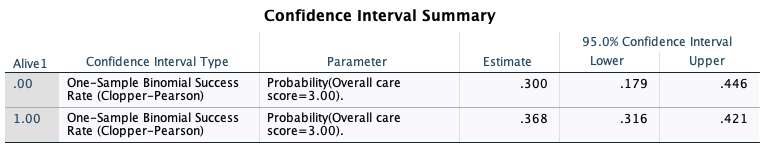


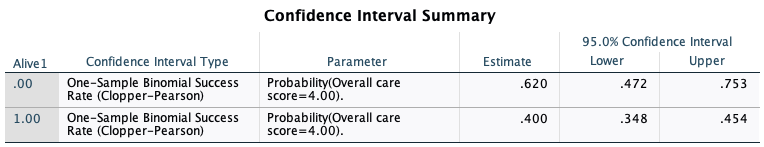


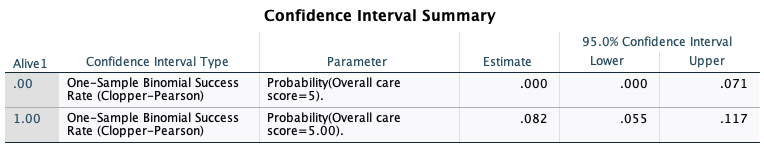


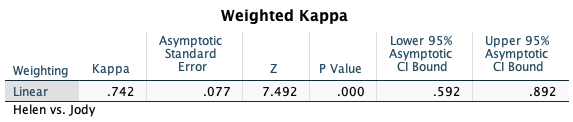


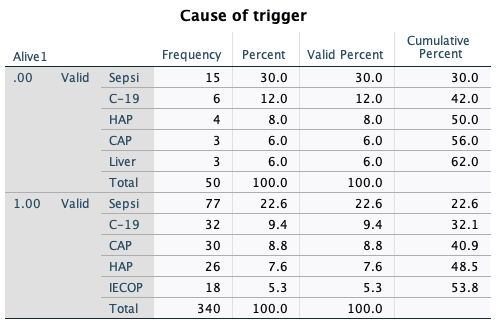


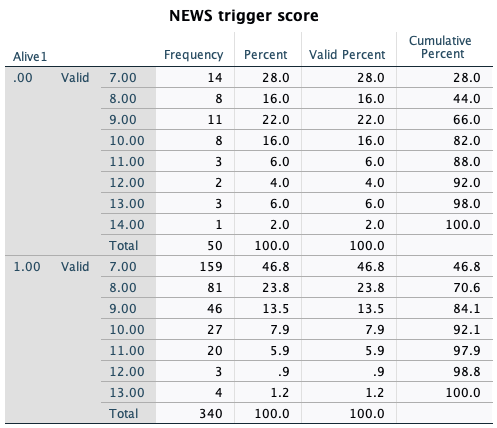


## Trigger Event Characteristics and Score Frequencies

Distribution of NEWS scores for patients first trigger of ≥7

| Trigger Score Frequencies n (%) | EWS ≥7 survivors  n=340 | EWS ≥7 non-survivors  n=50 |
| --- | --- | --- |
| 7  8  9  10  11  12  13  14 | 159 (46.8)  81 (23.8)  46 (13.5)  27 (7.9)  20 (5.9)  3 (0.9)  4 (1.2)  0 | 14 (28)  8 (16)  11 (22)  8 (16)  3 (6)  2 (4)  3 (6)  1 (2) |

## Escalation of care metrics comparing Survivors with Non-survivors


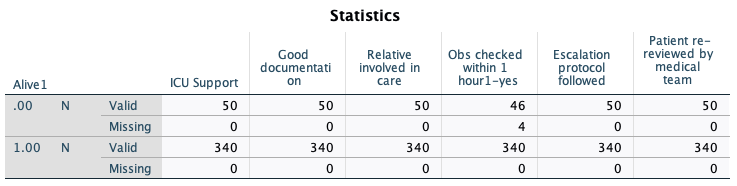


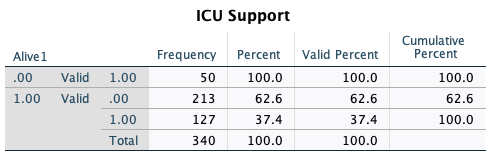


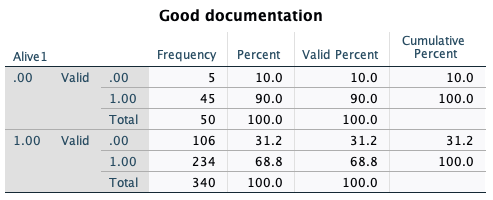


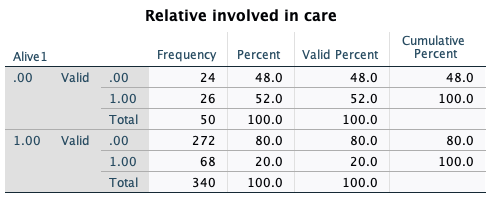


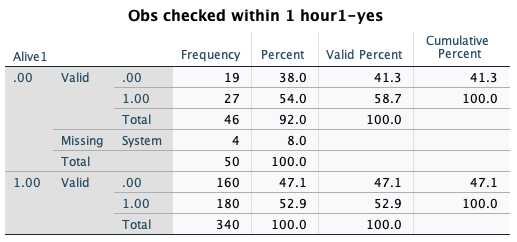


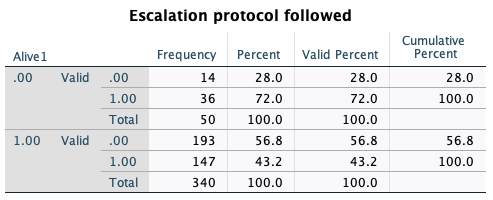


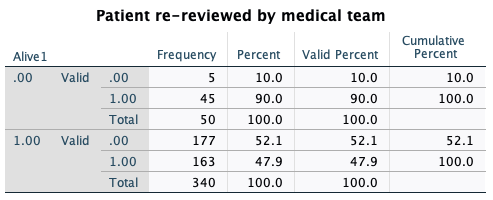


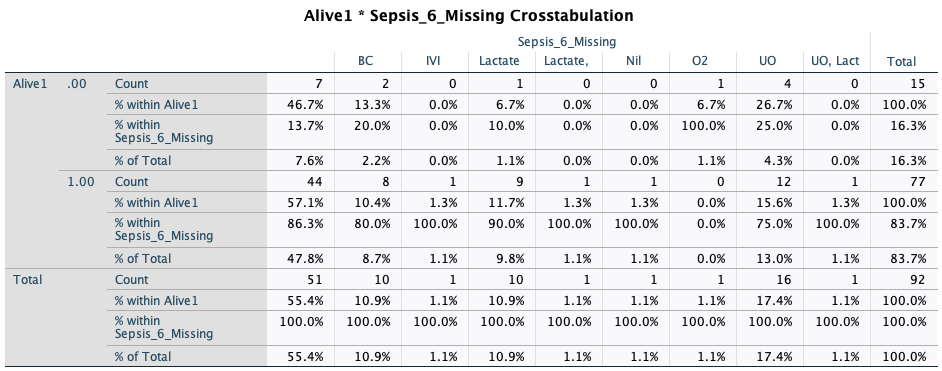


## Sub-Analysis by hospital Site in Survivors and Non-survivors


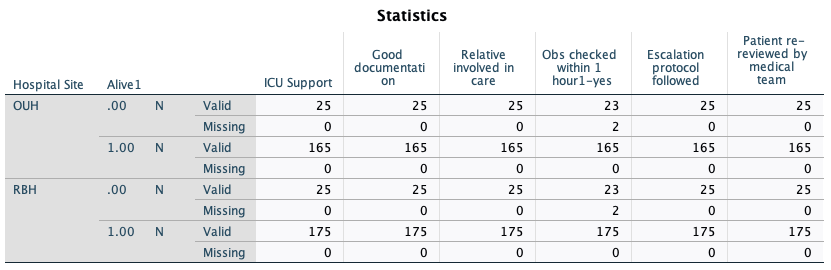


Site B

Site A


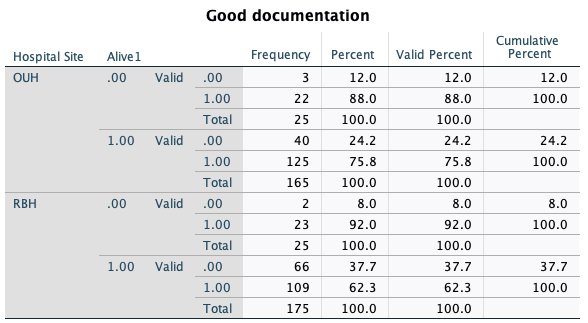

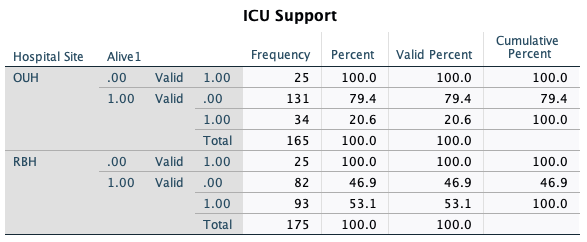


Site B

Site A

Site A

Site B


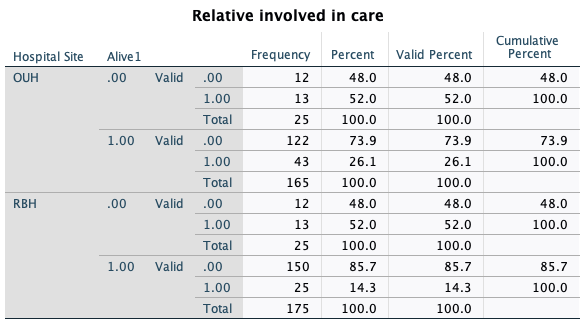


Site A

Site B


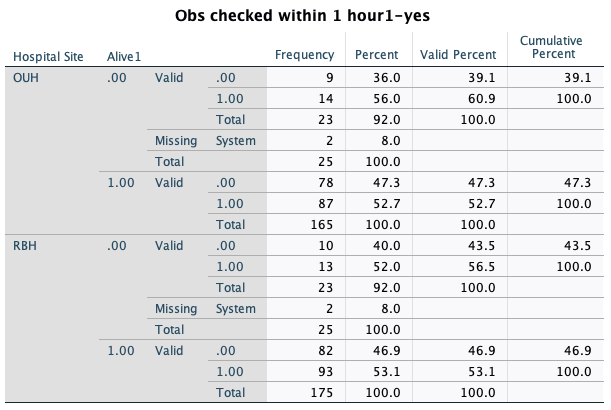


Site A

Site B


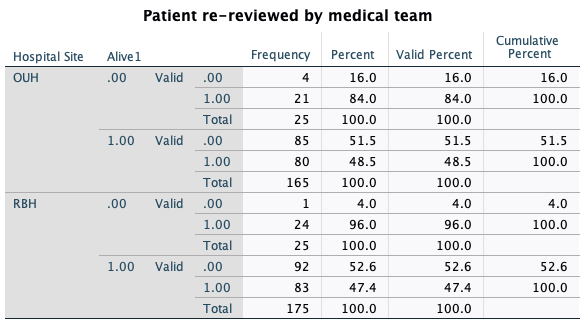


Site B

Site A
