# Supplementary material for: A retrospective records review comparing the care of patients who either avoided or were admitted to an ICU following a ward-based deterioration event
Source: Intensive Crit Care Nurs. 2025 Oct;90:None. doi: 10.1016/j.iccn.2025.104064 (PMC12396344; doi:10.1016/j.iccn.2025.104064)
Supplement: Supplementary Data 5 [file mmc5.docx]

Supplementary File 5 Prevalence of physiological parameters

| Physiological Parameter n (%) | Survivors | Non-survivors |
| --- | --- | --- |
| Temperature |  |  |
| Temp <36.1 | 18 (5.3) | 10 (20) |
| Temp >38 | 91(26.8) | 13 (26) |
| Heart Rate |  |  |
| Heart rate <51 | 3 (0.9) | 0 |
| Heart rate >90 | 165 (48.5) | 37 (74) |
| Respiratory Rate |  |  |
| Respiratory rate <12 | 4 (1.2) | 1 (2) |
| Respiratory rate >20 | 253 (74.4) | 42 (84) |
| Oxygen requirement | 226 (66.5) | 36 (72) |
| BP |  |  |
| Systolic BP <111 | 60 (17.6) | 24 (48) |
| Systolic BP <101 | 47 (13.5) | 17 (34) |
| Systolic BP <91 | 23 (6.8) | 9 (18) |
| Systolic BP >219 | 2 (0.6) | 0 |
| <A on AVPU | 36 (10.6) | 14 (28) |
| SpO2 |  |  |
| SpO2 >96 | 88 (25.9) | 10 (20) |
| SpO2 <95 | 252 (74.1) | 41 (82) |
| SpO2 <93 | 167 (49.1) | 28 (56) |
| Spo2 <91 | 97 (28.5) | 24 (48) |
